# Flavonoids with Anti-Herpes Simplex Virus Properties: Deciphering Their Mechanisms in Disrupting the Viral Life Cycle

**DOI:** 10.3390/v15122340

**Published:** 2023-11-29

**Authors:** Miroslava Šudomová, Sherif T. S. Hassan

**Affiliations:** 1 Museum of Literature in Moravia, Klášter 1, 664 61 Rajhrad, Czech Republic; sudomova@post.cz; 2 Department of Applied Ecology, Faculty of Environmental Sciences, Czech University of Life Sciences Prague, Kamýcká 129, 165 00 Prague, Czech Republic

**Keywords:** antiviral properties, cellular pathways, drug resistance, flavonoids, herpes simplex virus, host–virus interaction, HSV-1, HSV-2, HSV life cycle, natural antivirals, natural products

## Abstract

The herpes simplex virus (HSV) is a double-stranded DNA human virus that causes persistent infections with recurrent outbreaks. HSV exists in two forms: HSV-1, responsible for oral herpes, and HSV-2, primarily causing genital herpes. Both types can lead to significant complications, including neurological issues. Conventional treatment, involving acyclovir and its derivatives, faces challenges due to drug resistance. This underscores the imperative for continual research and development of new drugs, with a particular emphasis on exploring the potential of natural antivirals. Flavonoids have demonstrated promise in combating various viruses, including those within the herpesvirus family. This review, delving into recent studies, reveals the intricate mechanisms by which flavonoids decode their antiviral capabilities against HSV. By disrupting key stages of the viral life cycle, such as attachment to host cells, entry, DNA replication, latency, and reactivation, flavonoids emerge as formidable contenders in the ongoing battle against HSV infections.

## 1. Introduction

The herpes simplex virus (HSV) is an infectious human pathogen categorized within human herpesviruses as an alpha-herpesvirus. As a double-stranded DNA virus, it establishes a persistent infection in humans throughout their lifetime. HSV comprises two distinct species, namely HSV-1 and HSV-2 [1,2]. HSV-1 primarily leads to oral herpes through skin-to-skin contact and can also result in genital herpes through oral–genital contact. Genital herpes is commonly caused by the sexually transmitted HSV-2 virus, which can also infect the oral region [3,4]. Genital sores, often associated with HSV-2, can heighten the risk of both transmitting and acquiring other sexually transmitted infections, including the human immunodeficiency virus (HIV) [5,6]. Both types of HSV pose a risk of herpes disease in newborns and infants, often resulting in severe outcomes and substantial rates of mortality and morbidity [7].

From an epidemiological standpoint, the World Health Organization (WHO) approximates that globally, 3.7 billion individuals are affected by HSV-1, while 491 million are afflicted with HSV-2 [8]. HSV infection is predominantly contracted during childhood and is commonly transmitted through direct contact with an infected individual. Symptoms often go unnoticed, manifesting as asymptomatic viral shedding. Following the initial outbreak, infected persons may experience prodromal signs such as skin tingling, itching, or burning before the emergence of blisters. Given the persistent nature of HSV as a lifelong virus, multiple outbreaks can occur, with symptoms surfacing particularly when the immune system is compromised [9,10,11,12].

HSV is commonly managed with FDA-approved drugs, such as acyclovir and its derivatives. These medications function by inhibiting HSV DNA polymerase, a key enzyme in the replication process [13,14]. While effective in alleviating symptoms and shortening outbreaks, their overuse has led to drug resistance, compromising the overall efficacy of treatment. Additionally, HSV’s ability to establish latent infections in host cells adds complexity to antiviral approaches [15,16]. In the realm of anti-HSV research, scientists are actively seeking effective and natural remedies to counter HSV diseases. This quest has propelled investigations into the potential of flavonoids, derived from plants, as promising candidates [17,18].

Incorporating insights from recent studies, this review critically examines these compounds, evaluating their effectiveness in targeting various stages of the HSV life cycle. The ensuing comprehensive analysis within this review sheds light on their promising role in the ongoing battle against HSV infections.

We conducted an intensive literature search utilizing major online databases, including Web of Science Core Collection, Scopus, PubMed, SciFinder, ScienceDirect, Google Scholar, and ClinicalTrials.gov. Our search strategy involved employing specific keywords related to flavonoids with documented anti-HSV properties and elucidated mechanisms that target the viral life cycle. The collected data were sourced from studies published during the period spanning 2018 to 2023. To facilitate a thorough and nuanced evaluation and analysis, we have integrated select studies published before 2018.

## 2. A Brief Overview of the HSV Life Cycle

The HSV life cycle is a complex and dynamic process involving multiple stages. It typically commences with viral attachment and entry into host cells, specifically epithelial cells near mucous membranes or the skin [19,20,21]. This process is facilitated via viral glycoproteins interacting with cell surface receptors. Once inside the host cell, the virus releases its genetic material, consisting of double-stranded DNA, into the nucleus [22,23,24]. Subsequently, viral genes are transcribed and translated to produce new viral particles. The assembly of these particles takes place in the host cell’s cytoplasm, and mature virions are then transported to the cell membrane for release [25,26,27]. The virus can establish both lytic and latent infections. In a lytic infection, new virions are produced, leading to cell lysis and the release of viral progeny [28,29]. In contrast, during latent infection, the virus establishes a presence in sensory neurons, where the viral genome persists without active replication. Periodically, the virus may reactivate, leading to recurrent infections and the shedding of infectious particles [30,31]. Reactivation of the virus often occurs due to immunosuppression induced by various physiological and environmental influences that adversely affect the immune system [32,33]. The HSV life cycle is tightly regulated, involving intricate interactions between the virus and the cellular machinery of the host [34,35]. The ability of HSV to switch between lytic (active replication) and latent phases contributes to its persistence in the host. This complex life cycle provides multiple potential targets for antiviral interventions aimed at disrupting viral entry, replication, assembly, or release [36,37].

## 3. Bioactive Flavonoids: Nature’s Antiviral Arsenal

Plants synthesize flavonoids as secondary metabolites characterized by diverse chemical structures and biological functionalities [38]. Belonging to the polyphenol class, flavonoids represent a varied cohort of natural compounds ubiquitously distributed throughout the plant kingdom, contributing to the chromatic spectrum observed in fruits, vegetables, flowers, and beverages [39,40]. These compounds play a key role in plant physiology and developmental processes, as well as in conferring resistance against microbial and viral infections, UV radiation, and various abiotic stresses [41,42,43]. Chemically, flavonoids manifest as polyphenolic entities with a fundamental 15-carbon atom structure (C6-C3-C6), encompassing distinct subclasses such as flavones, protoflavones, isoflavones, flavanones, flavonols, flavanols (including catechins), anthocyanins/anthocyanidins, and chalcones [44,45]. The core flavonoid structure is shaped by the intricate interplay of hydroxylation, prenylation, and glycosylation. The abundance of these compounds in plants fluctuates based on agricultural and environmental conditions, with extraction methods further impacting the yielded amount [41,46].

Flavonoids have a rich history of contributing to human health through potent pharmacological actions, enhancing the immune system, and combating a variety of diseases [47]. Their formidable antiviral properties extend across both DNA and RNA viruses, employing diverse mechanisms to impede the virus replication process and potentially prevent infections [48,49]. Flavonoids exhibit the capability to disrupt multiple steps in the life cycles of various viruses and modulate the involved cellular pathways [50,51,52]. Based on these antiviral mechanisms, flavonoids are categorized as preventative inhibitors, therapeutic inhibitors, and immunomodulators [53].

## 4. Blocking HSV-1 Infection via Flavonoids

The promising advances in utilizing flavonoids against HSV-1 over the past five years have been substantiated through a multitude of laboratory and animal investigations. Among these studies, two methoxyflavones, namely 5,3′-dihydroxy-3,6,7,8,4′-pentamethoxyflavone (PMF) and 5-hydroxy-3,6,7,3′,4′-pentamethoxyflavone (PMF-OH), isolated from *Marcetia taxifolia*, were found to significantly inhibit HSV-1 activity by diminishing viral DNA replication [54].

Morusin, a prenylated flavone extracted from the young twig of *Morus alba* L. (Mori ramulus), inhibits HSV-1 DNA replication by targeting the synthesis of HSV-1 glycoprotein D (gD) and suppressing reactive oxygen species (ROS) induced by HSV-1. These dual mechanisms highlight its potential as a promising antiviral agent [55].

In a related experiment involving *Morus alba* L., Čulenová and coworkers [56] identified three prenylated flavonoids: kuwanon C, kuwanon T (flavones), and kuwanon U (flavanone). These compounds effectively impede HSV-1 multiplication, with molecular docking suggesting interference with HSV-1 DNA polymerase. A preliminary structure–activity relationship study features the potent efficacy of kuwanon T, attributed to its two prenyl units.

Wogonin, an active flavone from *Scutellaria baicalensis* Georgi, demonstrates a potent anti-HSV-1 effect in vitro. Its mechanisms include suppressing DNA replication, glycoprotein D (gD) mRNA transcription, and immediate-early (IE) gene expression [57].

In another research study using *Scutellaria baicalensis* Georgi, the flavone baicalein has proven effective in blocking HSV-1 replication, including acyclovir-resistant strains. Demonstrating efficacy in various models, it reduces viral loads, inflammation, and mortality in mice. Its dual mechanism, encompassing both viral particle inactivation and the inhibition of IκB kinase beta (IKK-β) phosphorylation, underscores its potential as a promising antiviral candidate against HSV-1 and its resistant forms [58].

Vitexin, a bioactive flavone from *Erythrina speciosa*, displays anti-HSV-1 activity by targeting the HSV-1 DNA polymerase, as revealed in laboratory tests and molecular docking analysis [59].

Luteolin, a natural flavone, efficiently combats drug-resistant strains and hinders early HSV-1 infection, showcasing potent antiviral properties. In both in vitro and in vivo experiments, it proved highly effective against herpes encephalitis (HSE). By activating the cyclic guanosine monophosphate–adenosine monophosphate synthase (cGAS)/stimulator of the interferon gene (STING) pathway, luteolin enhances interferon production, thereby impeding HSV-1 post-entry. Its dual impact involves blocking viral entry and bolstering the immune response [60].

Amentoflavone, a biflavonoid identified in *Ginkgo biloba* L., *Biophytum sensitivum*, and various *Garcinia* species, showcases robust antiviral efficacy against a spectrum of HSV-1 strains, including those exhibiting resistance. It adeptly disrupts the initial phases of HSV-1 infection through multifaceted mechanisms of action [61].

In a thorough investigation, myricetin, a dietary flavonol inherent in diverse vegetables and fruits, demonstrated significant antiviral capability against HSV-1 through multiple molecular mechanisms [62].

Yarmolinsky and colleagues [63] successfully isolated two flavonols, namely quercetin 3-*O*-rutinoside and quercetin 3-*O*-arabinoside, from *Phlomis viscosa* Poiret. Both compounds exhibit suppressive effects on HSV-1 infection, effectively restraining viral replication by diminishing the formation of viral plaques.

In another study, quercetin 3-*O*-rutinoside and kaempferol 3-*O*-rutinoside, both flavonols derived from *Lespedeza bicolor*, displayed their ability to combat HSV-1 through virucidal effects. Additionally, they induced efficacy in blocking viral infection by hampering the replication of viral DNA [64].

Another research team explored kaempferol-3-*O*-rhamnoside’s potential in treating HSE through a cell culture and mouse model. Their findings indicate that this flavonol reduces inflammation, inhibits viral-induced brain injury, and alleviates brain tissue damage in mice. It emerges as a promising therapeutic candidate for HSV-1-induced brain injury [65].

The flavonol isorhamnetin, present in *Ginkgo biloba*, significantly disrupted the initial infection of HSV-1 by hindering viral DNA replication [66].

Dihydromyricetin, alternatively recognized as ampelopsin, is a dihydroflavonol derived from *Ampelopsis grossedentata*. It effectively suppresses HSV-1 through diverse pathways, as supported by virological and biochemical analyses [67].

Biochanin A (BCA), an isoflavone extracted from *Trifolium pratense* L., displays significant anti-HSV-1 efficacy by inhibiting viral replication in vitro. In mouse models mimicking herpes simplex keratitis (HSK), the administration of BCA through eye drops not only reduces ocular lesions but also effectively suppresses HSV-1. These observations emphasize the potential therapeutic utility of BCA in the context of HSK treatment [68].

In an experiment exploring the impact of temperature on treating HSV-1 infection, the main catechin in *Camellia sinensis*, epigallocatechin gallate (EGCG), showed notable suppression of HSV-1 virions between 25 and 37 °C. While the study refrained from specifying the precise mechanism of action, the authors proposed that EGCG might disrupt various steps in the HSV-1 life cycle, drawing on prior research [69]. Meanwhile, a different research group studied EGCG’s effects on HSV-1 infection in oral epithelial cells, revealing confirmed mechanisms of action [70].

Wang et al. [71] illuminated the inhibitory potency of isoliquiritigenin, a chalcone-type compound, against HSV-1 replication. They revealed that the antiviral mechanism linked to isoliquiritigenin correlates with its agonistic impact on nuclear factor erythroid 2-related factor 2 (NRF2).

Vicente and colleagues [72] explored how cyanidin, an anthocyanin-type substance found in various berries, actively hinders HSV-1. They observed that this compound impedes viral adsorption and DNA replication, thereby blocking viral infection.

The anthocyanin delphinidin-3-glucoside chloride, also known as myrtillin, found in *Ribes nigrum* L. and *Vaccinium myrtillus* L., demonstrates anti-infectivity properties against HSV-1 by targeting viral DNA replication [73].

In a dual in vitro and in vivo study, the total flavonoids obtained from *Robinia pseudoacacia cv. idaho* impeded viral DNA replication in vitro, showing significant anti-HSV-1 activity. Additionally, researchers observed no adverse effects during the in vivo phase, affirming the safety of its potential practical applications [74].

In an in vivo experiment, the administration of total flavonoids from *Ixeris sonchifolia* (Bae.) Hance to mice with HSK significantly improved corneal lesions, reduced infection, and increased survival rates, underscoring its therapeutic potential [75].

**Figure 1:**
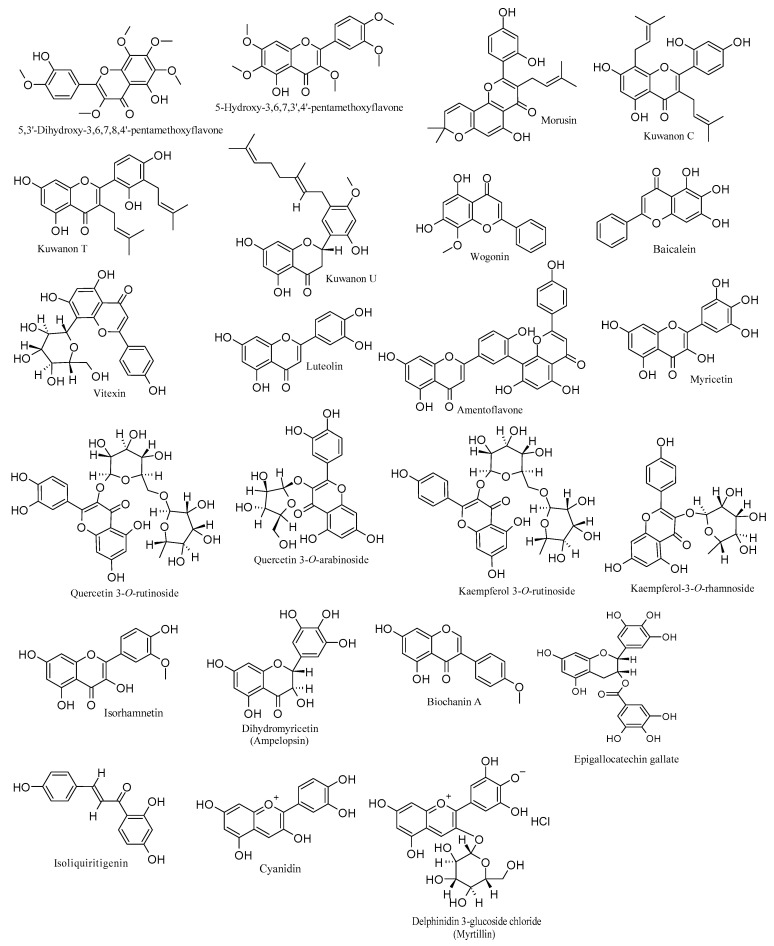
Chemical structures of flavonoids with anti-HSV-1 properties.

To sum up, the examined flavonoids, as discussed above, play a crucial role in interacting with different phases of the HSV-1 life cycle. They obstruct viral attachment, entry into target cells, DNA replication, and the expression of various viral genes and proteins. Consequently, they impede the virus’s capacity to infect, concurrently influencing essential cellular pathways integral to the viral life cycle. Table 1 details the anti-HSV-1 activities and mechanisms of flavonoids, complemented by Figure 1, which elucidates their chemical structures.

## 5. Blocking HSV-2 Infection via Flavonoids

In the last five years, there has been limited progress in investigating flavonoids in the context of HSV-2. Nevertheless, there is a clear emphasis on conducting experiments to delve into the mechanisms of action, reflecting a concentrated effort to understand how flavonoids may interact with the HSV-2 life cycle. In a laboratory setting, the flavone wogonin from *Scutellaria baicalensis* demonstrated significant inhibition of HSV-2 entry into Vero cells during the post-entry stage, resulting in a notable reduction in viral replication. Its mode of action has been clarified as targeting IE genes and gD expressions, as well as regulating cellular NF-κB and JNK/p38 MAPK pathways [57].

Flavones, specifically apigenin and luteolin, extracted from *Arisaema tortuosum*, were identified as inhibitors of HSV-2 replication, showing a reduction in viral progeny production [76].

The anti-HSV-2 activity of the flavonol myricetin was revealed through mechanisms targeting virus adsorption, membrane fusion, and DNA replication. Additionally, it affects several cellular pathways [62].

Isorhamnetin, a flavonol derived from *Ginkgo biloba*, markedly inactivated the primary infection of HSV-2 by inhibiting the replication of viral DNA [66].

The flavanone kuwanon E, extracted from *Morus alba*, exhibits the capacity to suppress HSV-2 replication in infected Vero cells. This antiviral effect is attributed to its interaction with the HSV-2 protease, as predicted with a molecular docking approach. Furthermore, a structure–activity relationship study associates its activity with the presence of a hydroxyl group at C-4′ [56].

Stamos et al. [77] revealed the antiherpetic efficacy of EGCG, derived from *Camellia sinensis*, against HSV-2. This compound impressively inhibits viral replication (99.9% inhibition), effectively impeding HSV-2 attachment to Vero cells by hindering glycoprotein D expression.

**Figure 2:**
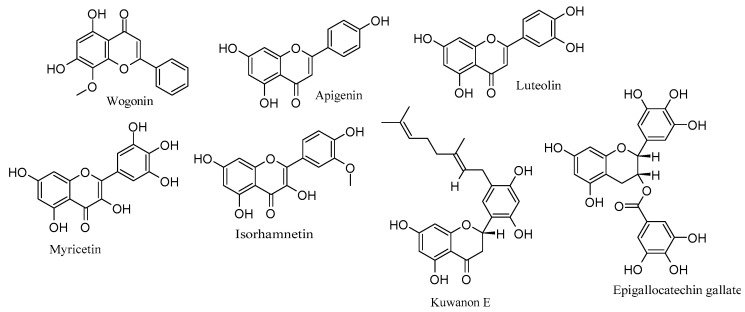
Chemical structures of flavonoids with anti-HSV-2 properties.

To recapitulate, flavonoids such as wogonin, apigenin, luteolin, myricetin, isorhamnetin, and kuwanon E exert notable anti-HSV-2 effects by targeting multiple stages of the viral life cycle and different cellular pathways. Additionally, EGCG stands out for its striking inhibition of HSV-2 replication. These findings emphasize the diverse and promising potential of flavonoids as effective anti-HSV-2 agents, acting through multifaceted mechanisms that involve interference with viral processes and modulation of cellular pathways. Table 2 outlines the anti-HSV-2 mechanisms of flavonoids, while Figure 2 highlights their respective chemical structures.

## 6. Flavonoid-Enhanced Approaches to Elevate HSV Therapy

### 6.1. Nanoparticles and Gel-Formulation-Based Strategies

Incorporating flavonoids into nanoparticles enhances drug delivery to HSV-affected cells by improving stability and bioavailability [78,79]. The nanoparticles boost drug solubility, prolong circulation time, and target specific cells, ensuring a focused approach that minimizes potential side effects in treating HSV diseases [80,81]. Elste et al. [82] conducted a study illustrating the potent antiviral capacity of plant cell-engineered gold nanoparticles conjugated with quercetin (pAuNPsQ) against HSV-1. These formulated nanoparticles effectively inhibit HSV-1 entry and replication through various mechanisms. Remarkably, pAuNPsQ shows promising outcomes in both pre-treating target cells and inducing virus neutralization. These findings underscore the potential of modifying plant cell-based nanomaterials with quercetin to develop cutting-edge antiviral formulations.

Another investigation explores EGCG-modified silver nanoparticles (EGCG-AgNPs) as a potential therapeutic intervention for herpes infections. These modified nanoparticles exhibit enhanced inhibition of the attachment and entry of both HSV-1 and HSV-2 in human keratinocytes compared to EGCG alone. In mouse models, EGCG-AgNPs significantly reduce virus titers and elicit a robust immune response in mucosal tissues, characterized by increased cell infiltration and elevated expression of key immune markers. These outcomes suggest EGCG-AgNPs as a promising dual-functioning antiviral intervention for mucosal applications [83].

Caldas Dos Santos et al. [84] harnessed C-glycosylflavonoids from *Cecropia glaziovii*, encapsulating them in PLGA nanoparticles. This formulation achieved 100% inhibition of HSV-1 replication with an IC_50_ value of 8.2 µg/mL, exhibiting no cytotoxicity on Vero cells. The study indicates the promising application of this preparation for effective HSV-1 infection therapy.

Gel-formulated polyphenols, including flavonoids, offer enhanced stability and controlled release, optimizing the delivery of antiviral compounds for the effective and targeted treatment of HSV infectivity [85]. Building on these advantages, an investigation into quercetin-loaded gels for treating HSV-1 revealed encouraging results. The poloxamer-based gel showed promise by efficiently restraining quercetin diffusion, exhibiting higher stability, and demonstrating a prolonged virucidal impact against the virus. These findings feature the potential of quercetin in a poloxamer-based gel as a favorable and precise treatment option for HSV-1 infection [85].

In the research conducted by Dickinson et al. [86], the PTV80 hand gel prototype, featuring EGCG-palmitate (EC16), revealed an efficacy exceeding 99.9% in reducing HSV-1 infectivity within a 60 s timeframe. These results highlight a robust and rapid virucidal effect against the virus, supporting the potential application of the hand gel in hand hygiene products to mitigate and manage outbreaks associated with this virus. The formulation’s non-toxic properties, as identified in the study, further enhance its suitability for widespread use in promoting hand hygiene and preventing infection transmission.

### 6.2. Physical Properties Targeting Approach

The regulation of herpesvirus infection is intricately tied to its molecular and physical properties. HSV medications, including acyclovir and its analogs, primarily target the molecular aspects of the virus, specifically focusing on viral proteins such as DNA polymerase [87,88]. Researchers have unveiled an innovative treatment targeting the physical properties of the HSV. This cutting-edge biophysical strategy adeptly mitigates the virus’s genome pressure, ensuring efficacy without compromising host cells. Functioning at an internal pressure of 20 atmospheres, the herpesvirus rapidly delivers genetic materials into the host cell nucleus upon entry. By finely adjusting this viral pressure, scientists have successfully hindered the virus’s propagation to other cells, thereby amplifying the effectiveness of antiherpetic drugs [89]. Considering this breakthrough, the synergistic potential between this strategy and anti-HSV drugs, including flavonoids, not only opens new possibilities for addressing HSV infections but also paves the way for a more nuanced understanding of antiviral approaches.

### 6.3. Combination Therapies

In recent years, scientific inquiry into the antiviral potential of flavonoids has expanded, shedding light on their synergistic effects when combined with conventional antiviral drugs. These combination therapies hold significant promise for enhancing efficacy and addressing some of the challenges associated with treating HSV infections, including drug resistance [48,90]. Wu and colleagues [70] investigated the combined impact of EGCG at 25 µg/mL and acyclovir at 50 µg/mL on HSV-1 infection in oral epithelial cells. Their findings revealed a significant inhibitory effect on HSV-1 replication, leading to a reduction in intracellular viral DNA at 20 h post-infection. Furthermore, the combined treatment repressed the expression of viral proteins ICP5 and gD, highlighting its potent antiviral efficacy. In a related study, the combined effects of wogonin and acyclovir in combating HSV-2 were explored using an in-cell western assay. The results indicated a moderate synergism (combination index (CI) = 0.8), suggesting that the combination could offer enhanced therapeutic benefits for HSV-2 treatment [57].

## 7. Clinical Studies

Over the last five years, challenges in studying flavonoids in clinical trials have been acknowledged, primarily due to constraints associated with conducting comprehensive investigations. However, a recent trial involving 68 individuals with oral herpes revealed promising results for the herbal blend Gene-Eden-VIR/Novirin. Enriched with quercetin (100 mg), the blend was administered daily for 2 to 36 months alongside standard protocols, significantly reducing the frequency and duration of outbreaks. Notably, it proved safer and more effective than traditional acyclovir and valacyclovir approaches [91].

In a preceding clinical study, the utilization of Gene-Eden-VIR/Novirin, containing 100 mg of quercetin, effectively diminished genital herpes infections in 90.8% of 139 participants over 2–48 months. The product exhibited superior efficacy and safety compared to standard antiviral drugs (acyclovir, valacyclovir, and famciclovir). These findings affirm that the product presents a favorable and well-tolerated choice for addressing genital herpes outbreaks, outperforming conventional treatments in clinical efficacy [92]. Moreover, this herbal product consistently showcased efficacy in curing both severe and mild cases of genital herpes infections, validated by a clinical study with an 87% success rate among 137 participants over 2–48 months [93].

## 8. Conclusions, Challenges, and Future Prospects

In conclusion, this review points out the robust potential of flavonoids as anti-HSV agents, emphasizing their antiviral efficacy across various stages of the viral life cycle. A synergy of in vitro, in vivo, and computational studies adds depth to the evidence supporting flavonoids’ multifaceted anti-HSV mechanism. By disrupting key molecular processes crucial for HSV propagation, flavonoids exhibit versatility in interfering with viral attachment, penetration, and replication. Additionally, they engage with critical cellular pathways integral to the viral life cycle, simultaneously boosting the immune system. Furthermore, their diverse structures, combined with the selective targeting of specific viral genes and proteins, position them as a prospective foundation for the development of novel anti-HSV drugs. This review also features strategies involving flavonoids to enhance HSV treatment.

However, the journey from promising research to mainstream anti-HSV treatments encounters intricate challenges. Limited bioavailability, attributed to issues such as poor solubility and rapid metabolism, complicates their efficacy. Achieving specificity to target HSV strains without adversely affecting host cells requires careful molecular design. Formulating flavonoids into stable delivery systems remains challenging, demanding innovations for enhanced solubility and controlled release. The structural complexity of flavonoids poses hurdles in synthesis and large-scale production, accompanied by cost implications. Concerns about viral resistance and the evolving HSV strains necessitate ongoing research. To overcome these challenges, future research should focus on improving bioavailability through novel delivery systems, conducting comprehensive structure-activity relationship studies, exploring combination therapies, and advancing genomic and proteomic approaches. Additionally, rigorous clinical trials and translational research are essential for bridging the gap between preclinical promise and practical applications, establishing flavonoids as a viable antiviral therapeutic option.

## Figures and Tables

**Table 1:** Flavonoids and their mechanisms in combatting the HSV-1 life cycle.

Compound, Chemical Classification, Concentration/Dose, and Source	Study Type, Strain, and Cell/Animal Models	Mechanisms of Action (Inhibition)	Reference
PMF and PMF-OH. Flavones. EC_50_ = 6.8 and 5.9 µM, respectively. *Marcetia taxifolia*.	In vitro. HSV-1. Vero cells.	DNA replication.	[54]
Morusin. Prenylated flavone. 20 µM. *Morus alba* L.	In vitro. HSV-1. Vero cells.	DNA replication. gD expression. HSV-1-induced ROS.	[55]
Kuwanon C and kuwanon T (prenylated flavones) and kuwanon U (prenylated flavanone). IC_50_ = 0.91, 0.64, and 1.93 µg/mL, respectively. *Morus alba* L.	In vitro and in silico. HSV-1. Vero cells.	DNA replication (in vitro). DNA polymerase (in silico).	[56]
Wogonin. Flavone. Various concentrations in µM. *Scutellaria baicalensis* Georgi.	In vitro. HSV-1. Vero cells.	DNA replication. IE genes expressions and gD mRNA transcription.	[57]
Baicalein. Flavone. Different concentrations in µM (in vitro). 200 mg/kg/day (in vivo). *Scutellaria baicalensis* Georgi.	In vitro and in vivo. HSV-1 and HSV-1 ACV-resistant strains. Vero and HaCat cells. BALB/c mice.	DNA replication, viral particles, IKK-β, and NF-κB (in vitro). Viral loads, inflammation, and mortality (in vivo).	[58]
Vitexin. Flavone. EC_50_ = 18 µg/mL. *Erythrina speciosa*.	In vitro and in silico. HSV-1 (clinical strain). Vero cells.	DNA replication (in vitro). Viral DNA polymerase (in silico).	[59]
Luteolin. Flavone. Different concentrations in µM (in vitro). 50 mg/kg (in vivo). Various fruits, vegetables, and medicinal plants.	In vitro and in vivo. HSV-1 and HSV-1 ACV-resistant strains. HaCaT, BV2, and Vero cells. HSE mouse models.	Viral entry and DNA replication (in vitro). Viral post-entry by activating the cGAS-STING pathway and IFN (in vivo).	[60]
Amentoflavone. Biflavonoid. EC_50_ values (22.1 to 25.7 µM). *Ginkgo biloba* L., *Biophytum sensitivum*, and *Garcinia* species.	In vitro. HSV-1 (F strain) and ACV-resistant strains (HSV-1/106, HSV-1/153, and HSV-1/Blue).	DNA replication. UL54, UL52, and UL27 expressions. ICP0 expression. Nuclear import of HSV-1.	[61]
Myricetin. Flavonol. (2.2–40 µM; in vitro) and (2.5 and 5 mg/kg; in vivo). Various vegetables and fruits.	In vitro and in vivo. HSV-1. Vero, HeLa, and Hep-2 cells. BALB/c mice.	Virus adsorption, membrane fusion, DNA replication, and gD synthesis (in vitro). Cellular EGFR/PI3K/Akt pathway (in vitro). Virus titers and DNA replication (in vivo).	[62]
Quercetin 3-*O*-rutinoside and quercetin 3-*O*-arabinoside. Flavonols. 2 µM and IC_50_ = 8.6 µM. *Phlomis viscosa* Poiret and *Lespedeza bicolor*.	In vitro. HSV-1 (KOS- and ACV-resistant strains) Vero cells.	Plaque formation. DNA replication. Virucidal effect.	[63,64]
Kaempferol 3-*O*-rutinoside. Flavonol. IC_50_ = 12.2 µM. *Lespedeza bicolor*.	In vitro. HSV-1. Vero cells.	DNA replication. Virucidal effect.	[64]
Kaempferol-3-*O*-rhamnoside. Flavonol. Various concentrations in µM (in vitro). Brain, liver, blood, and muscle samples with diverse treatments in µM (in vivo). Various medicinal herbs.	In vitro and in vivo. HSV-1. Vero cells. HSE mouse models.	Viral infection (in vitro). Viral-induced brain injury in HSE animal models (in vivo).	[65]
Isorhamnetin. Flavonol. IC_50_ = 8.37 µg/mL. *Ginkgo biloba*.	In vitro. HSV-1. A549 cell.	Initial infection. DNA replication.	[66]
Dihydromyricetin (ampelopsin). Dihydroflavonol. EC_50_ = 12.56 µM. 16–32 µM (gene expressions) *Ampelopsis grossedentata*.	In vitro. HSV-1. Vero cells.	Plaque formation and progeny virus production. DNA replication. IE genes (ICP4 and ICP22), early genes (ICP8 and UL42), and late genes (gB and VP1/2). mRNA-TLR9. NF-κB and TNFα pathways.	[67]
Biochanin A. Isoflavone. 50 to 150 µM. *Trifolium pratense* L.	In vitro and in vivo. HSV-1. Vero and HCECs cells. Male C57BL/6 mice	DNA replication (in vitro). IE, E, and L genes (in vitro). Apoptosis of the corneal epithelium of HSK-infected mice (in vivo).	[68]
Epigallocatechin gallate Flavanol (catechin). 1–2 µM at 25–37 °C and 25 µg/mL. *Camellia sinensis*.	In vitro. HSV-1. Vero and oral epithelial cells.	DNA replication in the viral entry phase. Viral particles. IE and ICP0 expressions.	[69,70]
Isoliquiritigenin. Chalcone. 25 and 50 µM. *Glycyrrhiza uralensis*.	In vitro. HSV-1. A549 cells.	DNA replication via a mechanism that links with its NRF2 agonistic action.	[71]
Cyanidin. Anthocyanin. EC_50_ = 4.6 µg/mL. Various types of berries.	In vitro. HSV-1 ACV-resistant strain. HCLE cells.	Viral adsorption. DNA replication.	[72]
Delphinidin-3-glucoside chloride (myrtillin). Anthocyanin. 150 µg/mL.	In vitro. HSV-1. Vero cells.	DNA replication.	[73]
Total flavonoids. 0.46 g/mL (in vitro). 0.3 g/day/4 weeks (in vivo). *Robinia pseudoacacia cv. Idaho*.	In vitro and in vivo. HSV-1. Vero cells. Wistar rats.	DNA replication.	[74]
Total flavonoids. 50, 100, and 200 mg/kg (twice a day for 14 days). *Ixeris Sonchifolia* (Bae.) Hance.	In vivo. HSV-1. HSK-BALB/c mice.	Viral infection. IL-4 levels in the serum of mice.	[75]

Abbreviations: ACV, acyclovir; A549 cells, human alveolar type II epithelial cells; Akt, protein kinase B; BV2 cells, mouse microglia cells; cGAS, cyclic guanosine monophosphate–adenosine monophosphate synthase; DNA, deoxyribonucleic acid; EGFR, epidermal growth factor receptor; EC_50_, 50% effective concentration; gB, glycoprotein B; gD, glycoprotein D; HaCat cells, human keratinocytes cells HCECs, human corneal epithelial cells; HCLE cells, human corneal-limbal epithelial cells; HSK, herpes simplex keratitis; HSV-1, herpes simplex virus type 1; HSE, herpes simplex virus encephalitis; IC_50_, 50% inhibitory concentration; IE genes, immediate-early genes; ICP, infected cell protein; IKK-β, IκB kinase beta; IL-4, interleukin-4; IFN, interferon; KOS, an acyclovir-susceptible strain; L, late gene; mRNA, messenger ribonucleic acid; NF-κB, nuclear factor-κB; NRF2, nuclear factor erythroid 2-related factor 2; PI3K, phosphoinositide-3-kinase; PMF, 5,3′-dihydroxy-3,6,7,8,4′-pentamethoxyflavone; PMF-OH, 5-hydroxy-3,6,7,3′,4′-pentamethoxyflavone; ROS, reactive oxygen species; STING, stimulator of interferon gene; TLR9, Toll-like receptor 9; TNFα, tumor necrosis factor-α; UL27, late gene; UL52, early gene; UL54, viral immediate early gene; and Vero cells, African green monkey kidney cells.

**Table 2:** Flavonoids and their mechanisms in targeting the HSV-2 life cycle.

Compound, Chemical Classification, Concentration/Dose, and Source	Study Type, Strain, and Cell/Animal Models	Mechanisms of Action (Inhibition)	Reference
Wogonin. Flavone. Various concentrations in µM. *Scutellaria baicalensis* Georgi.	In vitro. HSV-2. Vero and HEC-1-A cells.	DNA replication. Viral protein synthesis. HSV-2 virions. IE and gD expressions. mRNA transcription. Cellular NF-κB and JNK/p38 MAPK pathways.	[57]
Apigenin and luteolin. Flavones. EC_50_ = 0.05 and 0.41 µg/mL, respectively. *Arisaema tortuosum*.	In vitro. HSV-2 and ACV-resistant HSV-2. Vero cells.	DNA replication. Viral progeny production. Cell-to-cell virus spread.	[76]
Myricetin. Flavonol. 2.2–40 µM. Various vegetables and fruits.	In vitro and in silico. HSV-2. Vero, HeLa, and Hep-2 cells.	Virus adsorption, membrane fusion, and DNA replication (in vitro). Cellular EGFR/PI3K/Akt pathway. HSV-2 gD (in silico).	[62]
Isorhamnetin. Flavonol. IC_50_ = 7.08 µg/mL. *Ginkgo biloba*.	In vitro. HSV-2. A549 cell.	Primary infection. DNA replication.	[66]
Kuwanon E. Prenylated flavanone. EC_50_ = 1.61 µg/mL. *Morus alba* L.	In vitro and in silico. HSV-2. Vero cells.	DNA replication (in vitro). HSV-2 protease (in silico).	[56]
Epigallocatechin gallate. Flavanol (catechin). 75 µM (99.9% inhibition). *Camellia sinensis*.	In vitro. HSV-2. Vero cells.	Viral attachment. DNA replication. gD expression.	[77]

Abbreviations: ACV, acyclovir; Akt, protein kinase B; DNA, deoxyribonucleic acid; EC_50_, 50% effective concentration; EGFR, epidermal growth factor receptor; gD, glycoprotein D; HEC-1-A cells, human endometrial cells; HSV-2, herpes simplex virus type 2; IC_50_, 50% inhibitory concentration; IE genes, immediate-early genes; IL-4, interleukin-4; JNK, c-Jun N-terminal kinase; mRNA, messenger ribonucleic acid; NF-κB, nuclear factor-κB; p38 MAPK, p38 mitogen-activated protein kinase; PI3K, phosphoinositide-3-kinase; and Vero cells, African green monkey kidney cells.

## Data Availability

The data presented in the manuscript encompass all relevant information.

## References

[1] Tognarelli E.I., Palomino T.F., Corrales N., Bueno S.M., Kalergis A.M., González P.A. (2019). Herpes Simplex Virus Evasion of Early Host Antiviral Responses. Front. Cell. Infect. Microbiol..

[2] Brezáni V., Leláková V., Hassan S.T.S., Berchová-Bímová K., Nový P., Klouček P., Maršík P., Dall’Acqua S., Hošek J., Šmejkal K. (2018). Anti-Infectivity against Herpes Simplex Virus and Selected Microbes and Anti-Inflammatory Activities of Compounds Isolated from *Eucalyptus globulus* Labill. Viruses.

[3] Omarova S., Cannon A., Weiss W., Bruccoleri A., Puccio J. (2022). Genital Herpes Simplex Virus—An Updated Review. Adv. Pediatr..

[4] Petti S., Lodi G. (2019). The Controversial Natural History of Oral Herpes Simplex Virus Type 1 Infection. Oral Dis..

[5] Hendrickx D.M., Sousa J.D., Libin P.J.K., Delva W., Liesenborgs J., Hens N., Müller V., Vandamme A.-M. (2021). Comparison of Two Simulators for Individual Based Models in HIV Epidemiology in a Population with HSV 2 in Yaoundé (Cameroon). Sci. Rep..

[6] Desai D.V., Kulkarni S.S. (2015). Herpes Simplex Virus: The Interplay Between HSV, Host, and HIV-1. Viral Immunol..

[7] Pinninti S.G., Kimberlin D.W. (2018). Neonatal Herpes Simplex Virus Infections. Semin. Perinatol..

[8] Herpes Simplex Virus. https://www.who.int/news-room/fact-sheets/detail/herpes-simplex-virus.

[9] Alareeki A., Osman A.M.M., Khandakji M.N., Looker K.J., Harfouche M., Abu-Raddad L.J. (2023). Epidemiology of Herpes Simplex Virus Type 2 in Europe: Systematic Review, Meta-Analyses, and Meta-Regressions. Lancet Reg. Health—Eur..

[10] Samies N.L., James S.H. (2020). Prevention and Treatment of Neonatal Herpes Simplex Virus Infection. Antiviral Res..

[11] Fatahzadeh M., Schwartz R.A. (2007). Human Herpes Simplex Virus Infections: Epidemiology, Pathogenesis, Symptomatology, Diagnosis, and Management. J. Am. Acad. Dermatol..

[12] Kurt-Jones E.A., Orzalli M.H., Knipe D.M. (2017). Innate Immune Mechanisms and Herpes Simplex Virus Infection and Disease. Adv. Anat. Embryol. Cell Biol..

[13] Poole C.L., James S.H. (2018). Antiviral Therapies for Herpesviruses: Current Agents and New Directions. Clin. Ther..

[14] Schalkwijk H.H., Snoeck R., Andrei G. (2022). Acyclovir Resistance in Herpes Simplex Viruses: Prevalence and Therapeutic Alternatives. Biochem. Pharmacol..

[15] Piret J., Boivin G. (2011). Resistance of Herpes Simplex Viruses to Nucleoside Analogues: Mechanisms, Prevalence, and Management. Antimicrob. Agents Chemother..

[16] Hassan S.T.S., Šudomová M., Berchová-Bímová K., Šmejkal K., Echeverría J. (2019). Psoromic Acid, a Lichen-Derived Molecule, Inhibits the Replication of HSV-1 and HSV-2, and Inactivates HSV-1 DNA Polymerase: Shedding Light on Antiherpetic Properties. Molecules.

[17] Jiang Y.-C., Feng H., Lin Y.-C., Guo X.-R. (2016). New Strategies against Drug Resistance to Herpes Simplex Virus. Int. J. Oral Sci..

[18] Ruchawapol C., Yuan M., Wang S.-M., Fu W.-W., Xu H.-X. (2021). Natural Products and Their Derivatives against Human Herpesvirus Infection. Molecules.

[19] Cairns T.M., Connolly S.A. (2021). Entry of Alphaherpesviruses. Curr. Issues Mol. Biol..

[20] Agelidis A.M., Shukla D. (2015). Cell Entry Mechanisms of HSV: What We Have Learned in Recent Years. Future Virol..

[21] Zhu S., Viejo-Borbolla A. (2021). Pathogenesis and Virulence of Herpes Simplex Virus. Virulence.

[22] Azab W., Osterrieder K. (2017). Initial Contact: The First Steps in Herpesvirus Entry. Adv. Anat. Embryol. Cell Biol..

[23] Connolly S.A., Jardetzky T.S., Longnecker R. (2021). The Structural Basis of Herpesvirus Entry. Nat. Rev. Microbiol..

[24] Arii J., Kawaguchi Y. (2018). The Role of HSV Glycoproteins in Mediating Cell Entry. Adv. Exp. Med. Biol..

[25] Heming J.D., Conway J.F., Homa F.L. (2017). Herpesvirus Capsid Assembly and DNA Packaging. Adv. Anat. Embryol. Cell Biol..

[26] Adlakha M., Livingston C.M., Bezsonova I., Weller S.K. (2020). The Herpes Simplex Virus 1 Immediate Early Protein ICP22 Is a Functional Mimic of a Cellular J Protein. J. Virol..

[27] Adler B., Sattler C., Adler H. (2017). Herpesviruses and Their Host Cells: A Successful Liaison. Trends Microbiol..

[28] Krawczyk E., Kangas C., He B. (2023). HSV Replication: Triggering and Repressing STING Functionality. Viruses.

[29] Rice S.A. (2021). Release of HSV-1 Cell-Free Virions: Mechanisms, Regulation, and Likely Role in Human-Human Transmission. Viruses.

[30] Cohen J.I. (2020). Herpesvirus Latency. J. Clin. Investig..

[31] Lomonte P. (2017). Herpesvirus Latency: On the Importance of Positioning Oneself. Adv. Anat. Embryol. Cell Biol..

[32] Ostler J.B., Sawant L., Harrison K., Jones C. (2021). Regulation of Neurotropic Herpesvirus Productive Infection and Latency-Reactivation Cycle by Glucocorticoid Receptor and Stress-Induced Transcription Factors. Vitam. Horm..

[33] Reese T.A. (2016). Coinfections: Another Variable in the Herpesvirus Latency-Reactivation Dynamic. J. Virol..

[34] Harrison K.S., Jones C. (2022). Regulation of Herpes Simplex Virus Type 1 Latency-Reactivation Cycle and Ocular Disease by Cellular Signaling Pathways. Exp. Eye Res..

[35] Asha K., Sharma-Walia N. (2021). Targeting Host Cellular Factors as a Strategy of Therapeutic Intervention for Herpesvirus Infections. Front. Cell. Infect. Microbiol..

[36] Kukhanova M.K., Korovina A.N., Kochetkov S.N. (2014). Human Herpes Simplex Virus: Life Cycle and Development of Inhibitors. Biochemistry.

[37] Packard J.E., Dembowski J.A. (2021). HSV-1 DNA Replication-Coordinated Regulation by Viral and Cellular Factors. Viruses.

[38] Wen L., Jiang Y., Yang J., Zhao Y., Tian M., Yang B. (2017). Structure, Bioactivity, and Synthesis of Methylated Flavonoids. Ann. N. Y. Acad. Sci..

[39] Chen L., Cao H., Huang Q., Xiao J., Teng H. (2022). Absorption, Metabolism and Bioavailability of Flavonoids: A Review. Crit. Rev. Food Sci. Nutr..

[40] Safe S., Jayaraman A., Chapkin R.S., Howard M., Mohankumar K., Shrestha R. (2021). Flavonoids: Structure-Function and Mechanisms of Action and Opportunities for Drug Development. Toxicol. Res..

[41] Liu W., Feng Y., Yu S., Fan Z., Li X., Li J., Yin H. (2021). The Flavonoid Biosynthesis Network in Plants. Int. J. Mol. Sci..

[42] Petrussa E., Braidot E., Zancani M., Peresson C., Bertolini A., Patui S., Vianello A. (2013). Plant Flavonoids--Biosynthesis, Transport and Involvement in Stress Responses. Int. J. Mol. Sci..

[43] Šamec D., Karalija E., Šola I., Vujčić Bok V., Salopek-Sondi B. (2021). The Role of Polyphenols in Abiotic Stress Response: The Influence of Molecular Structure. Plants.

[44] Teng H., Chen L. (2019). Polyphenols and Bioavailability: An Update. Crit. Rev. Food Sci. Nutr..

[45] Hassan S.T.S., Šudomová M. (2022). Molecular Mechanisms of Flavonoids against Tumor Gamma-Herpesviruses and Their Correlated Cancers—A Focus on EBV and KSHV Life Cycles and Carcinogenesis. Int. J. Mol. Sci..

[46] Singh B., Kumar A., Malik A.K. (2017). Flavonoids Biosynthesis in Plants and Its Further Analysis by Capillary Electrophoresis. Electrophoresis.

[47] Wen K., Fang X., Yang J., Yao Y., Nandakumar K.S., Salem M.L., Cheng K. (2021). Recent Research on Flavonoids and Their Biomedical Applications. Curr. Med. Chem..

[48] Šudomová M., Berchová-Bímová K., Mazurakova A., Šamec D., Kubatka P., Hassan S.T.S. (2022). Flavonoids Target Human Herpesviruses That Infect the Nervous System: Mechanisms of Action and Therapeutic Insights. Viruses.

[49] Russo M., Moccia S., Spagnuolo C., Tedesco I., Russo G.L. (2020). Roles of Flavonoids against Coronavirus Infection. Chem. Biol. Interact..

[50] Sharma V., Sehrawat N., Sharma A., Yadav M., Verma P., Sharma A.K. (2021). Multifaceted Antiviral Therapeutic Potential of Dietary Flavonoids: Emerging Trends and Future Perspectives. Biotechnol. Appl. Biochem..

[51] Ninfali P., Antonelli A., Magnani M., Scarpa E.S. (2020). Antiviral Properties of Flavonoids and Delivery Strategies. Nutrients.

[52] Hassan S.T.S., Masarčíková R., Berchová K. (2015). Bioactive Natural Products with Anti-Herpes Simplex Virus Properties. J. Pharm. Pharmacol..

[53] Zakaryan H., Arabyan E., Oo A., Zandi K. (2017). Flavonoids: Promising Natural Compounds against Viral Infections. Arch. Virol..

[54] Ortega J.T., Serrano M.L., Suárez A.I., Baptista J., Pujol F.H., Cavallaro L.V., Campos H.R., Rangel H.R. (2019). Antiviral Activity of Flavonoids Present in Aerial Parts of Marcetia Taxifolia against Hepatitis B Virus, Poliovirus, and Herpes Simplex Virus in Vitro. EXCLI J..

[55] Kim T.I., Kwon E.-B., Oh Y.-C., Go Y., Choi J.-G. (2021). Mori Ramulus and Its Major Component Morusin Inhibit Herpes Simplex Virus Type 1 Replication and the Virus-Induced Reactive Oxygen Species. Am. J. Chin. Med..

[56] Čulenová M., Sychrová A., Hassan S.T.S., Berchová-Bímová K., Svobodová P., Helclová A., Michnová H., Hošek J., Vasilev H., Suchý P. (2020). Multiple In Vitro Biological Effects of Phenolic Compounds from *Morus alba* Root Bark. J. Ethnopharmacol..

[57] Chu Y., Lv X., Zhang L., Fu X., Song S., Su A., Chen D., Xu L., Wang Y., Wu Z. (2020). Wogonin Inhibits in Vitro Herpes Simplex Virus Type 1 and 2 Infection by Modulating Cellular NF-κB and MAPK Pathways. BMC Microbiol..

[58] Luo Z., Kuang X.-P., Zhou Q.-Q., Yan C.-Y., Li W., Gong H.-B., Kurihara H., Li W.-X., Li Y.-F., He R.-R. (2020). Inhibitory Effects of Baicalein against Herpes Simplex Virus Type 1. Acta Pharm. Sin. B.

[59] Fahmy N.M., Al-Sayed E., Moghannem S., Azam F., El-Shazly M., Singab A.N. (2020). Breaking Down the Barriers to a Natural Antiviral Agent: Antiviral Activity and Molecular Docking of Erythrina Speciosa Extract, Fractions, and the Major Compound. Chem. Biodivers..

[60] Wang Y., Li F., Wang Z., Song X., Ren Z., Wang X., Wang Y., Zheng K. (2023). Luteolin Inhibits Herpes Simplex Virus 1 Infection by Activating Cyclic Guanosine Monophosphate-Adenosine Monophosphate Synthase-Mediated Antiviral Innate Immunity. Phytomedicine.

[61] Li F., Song X., Su G., Wang Y., Wang Z., Jia J., Qing S., Huang L., Wang Y., Zheng K. (2019). Amentoflavone Inhibits HSV-1 and ACV-Resistant Strain Infection by Suppressing Viral Early Infection. Viruses.

[62] Li W., Xu C., Hao C., Zhang Y., Wang Z., Wang S., Wang W. (2020). Inhibition of Herpes Simplex Virus by Myricetin through Targeting Viral gD Protein and Cellular EGFR/PI3K/Akt Pathway. Antivir. Res..

[63] Yarmolinsky L., Nakonechny F., Budovsky A., Zeigerman H., Khalfin B., Sharon E., Yarmolinsky L., Ben-Shabat S., Nisnevitch M. (2023). Antimicrobial and Antiviral Compounds of Phlomis Viscosa Poiret. Biomedicines.

[64] Tarbeeva D.V., Krylova N.V., Iunikhina O.V., Likhatskaya G.N., Kalinovskiy A.I., Grigorchuk V.P., Shchelkanov M.Y., Fedoreyev S.A. (2022). Biologically Active Polyphenolic Compounds from *Lespedeza bicolor*. Fitoterapia.

[65] Zhao C., Wang F., Tang B., Han J., Li X., Lian G., Li X., Hao S. (2021). Anti-Inflammatory Effects of Kaempferol-3-O-Rhamnoside on HSV-1 Encephalitis in Vivo and in Vitro. Neurosci. Lett..

[66] Sochocka M., Sobczyński M., Ochnik M., Zwolińska K., Leszek J. (2019). Hampering Herpesviruses HHV-1 and HHV-2 Infection by Extract of Ginkgo Biloba (EGb) and Its Phytochemical Constituents. Front. Microbiol..

[67] Zhou H.-Y., Gao S.-Q., Gong Y.-S., Lin T., Tong S., Xiong W., Shi C.-Y., Wang W.-Q., Fang J.-G. (2020). Anti-HSV-1 Effect of Dihydromyricetin from *Ampelopsis grossedentata* via the TLR9-Dependent Anti-Inflammatory Pathway. J. Glob. Antimicrob. Resist..

[68] Zhou N., Zheng D., You Q., Chen T., Jiang J., Shen W., Zhang D., Liu J., Chen D., Hu K. (2023). Therapeutic Potential of Biochanin A in Herpes Simplex Keratitis. Pharmaceuticals.

[69] Pradhan P., Nguyen M.L. (2018). Herpes Simplex Virus Virucidal Activity of MST-312 and Epigallocatechin Gallate. Virus Res..

[70] Wu C.-Y., Yu Z.-Y., Chen Y.-C., Hung S.-L. (2021). Effects of Epigallocatechin-3-Gallate and Acyclovir on Herpes Simplex Virus Type 1 Infection in Oral Epithelial Cells. J. Formos. Med. Assoc..

[71] Wang H., Jia X., Zhang M., Cheng C., Liang X., Wang X., Xie F., Wang J., Yu Y., He Y. (2023). Isoliquiritigenin Inhibits Virus Replication and Virus-Mediated Inflammation via NRF2 Signaling. Phytomedicine.

[72] Vicente J., Benedetti M., Martelliti P., Vázquez L., Gentilini M.V., Peñaranda Figueredo F.A., Nabaes Jodar M.S., Viegas M., Barquero A.A., Bueno C.A. (2023). The Flavonoid Cyanidin Shows Immunomodulatory and Broad-Spectrum Antiviral Properties, Including SARS-CoV-2. Viruses.

[73] Sivarajan R., Oberwinkler H., Roll V., König E.-M., Steinke M., Bodem J. (2022). A Defined Anthocyanin Mixture Sourced from Bilberry and Black Currant Inhibits Measles Virus and Various Herpesviruses. BMC Complement. Med. Ther..

[74] Guo H., Wan X., Niu F., Sun J., Shi C., Ye J.M., Zhou C. (2019). Evaluation of Antiviral Effect and Toxicity of Total Flavonoids Extracted from *Robinia pseudoacacia* Cv. Idaho. Biomed. Pharmacother..

[75] Wang Y.-Q., Cai L., Zhang N., Zhang J., Wang H.-H., Zhu W. (2020). Protective Effect of Total Flavonoids from Ixeris Sonchifolia on Herpes Simplex Virus Keratitis in Mice. BMC Complement. Med. Ther..

[76] Rittà M., Marengo A., Civra A., Lembo D., Cagliero C., Kant K., Lal U.R., Rubiolo P., Ghosh M., Donalisio M. (2020). Antiviral Activity of a *Arisaema tortuosum* Leaf Extract and Some of Its Constituents against Herpes Simplex Virus Type 2. Planta Med..

[77] Stamos J.D., Lee L.H., Taylor C., Elias T., Adams S.D. (2022). In Vitro and In Silico Analysis of the Inhibitory Activity of EGCG-Stearate against Herpes Simplex Virus-2. Microorganisms.

[78] Obisesan O., Katata-Seru L., Mufamadi S., Mufhandu H. (2021). Applications of Nanoparticles for Herpes Simplex Virus (HSV) and Human Immunodeficiency Virus (HIV) Treatment. J. Biomed. Nanotechnol..

[79] Treml J., Gazdová M., Šmejkal K., Šudomová M., Kubatka P., Hassan S.T.S. (2020). Natural Products-Derived Chemicals: Breaking Barriers to Novel Anti-HSV Drug Development. Viruses.

[80] Tomaszewska E., Ranoszek-Soliwoda K., Bednarczyk K., Lech A., Janicka M., Chodkowski M., Psarski M., Celichowski G., Krzyzowska M., Grobelny J. (2022). Anti-HSV Activity of Metallic Nanoparticles Functionalized with Sulfonates vs. Polyphenols. Int. J. Mol. Sci..

[81] Paradowska E., Studzińska M., Jabłońska A., Lozovski V., Rusinchuk N., Mukha I., Vitiuk N., Leśnikowski Z.J. (2021). Antiviral Effect of Nonfunctionalized Gold Nanoparticles against Herpes Simplex Virus Type-1 (HSV-1) and Possible Contribution of Near-Field Interaction Mechanism. Molecules.

[82] Elste J., Kumari S., Sharma N., Razo E.P., Azhar E., Gao F., Nunez M.C., Anwar W., Mitchell J.C., Tiwari V. (2023). Plant Cell-Engineered Gold Nanoparticles Conjugated to Quercetin Inhibit SARS-CoV-2 and HSV-1 Entry. Int. J. Mol. Sci..

[83] Krzyzowska M., Janicka M., Chodkowski M., Patrycy M., Obuch-Woszczatyńska O., Tomaszewska E., Ranoszek-Soliwoda K., Celichowski G., Grobelny J. (2023). Epigallocatechin Gallate-Modified Silver Nanoparticles Show Antiviral Activity against Herpes Simplex Type 1 and 2. Viruses.

[84] Caldas Dos Santos T., Rescignano N., Boff L., Reginatto F.H., Simões C.M.O., de Campos A.M., Mijangos C. (2017). In Vitro Antiherpes Effect of C-Glycosyl Flavonoid Enriched Fraction of *Cecropia glaziovii* Encapsulated in PLGA Nanoparticles. Mater. Sci. Eng. C Mater. Biol. Appl..

[85] Sicurella M., Sguizzato M., Mariani P., Pepe A., Baldisserotto A., Buzzi R., Huang N., Simelière F., Burholt S., Marconi P. (2022). Natural Polyphenol-Containing Gels against HSV-1 Infection: A Comparative Study. Nanomaterials.

[86] Dickinson D., Marsh B., Shao X., Liu E., Sampath L., Yao B., Jiang X., Hsu S. (2022). Virucidal Activities of Novel Hand Hygiene and Surface Disinfectant Formulations Containing EGCG-Palmitates (EC16). Am. J. Infect. Control.

[87] Brandariz-Nuñez A., Liu T., Du T., Evilevitch A. (2019). Pressure-Driven Release of Viral Genome into a Host Nucleus Is a Mechanism Leading to Herpes Infection. Elife.

[88] Bauer D.W., Huffman J.B., Homa F.L., Evilevitch A. (2013). Herpes Virus Genome, the Pressure Is on. J. Am. Chem. Soc..

[89] Brandariz-Nuñez A., Robinson S.J., Evilevitch A. (2020). Pressurized DNA State inside Herpes Capsids—A Novel Antiviral Target. PLoS Pathog..

[90] Šudomová M., Hassan S.T.S. (2021). Nutraceutical Curcumin with Promising Protection against Herpesvirus Infections and Their Associated Inflammation: Mechanisms and Pathways. Microorganisms.

[91] Polansky H., Javaherian A., Itzkovitz E. (2018). Clinical Trial of Herbal Treatment Gene-Eden-VIR/Novirin in Oral Herpes. J. Evid. Based Integr. Med..

[92] Polansky H., Javaherian A., Itzkovitz E. (2016). Clinical Study in Genital Herpes: Natural Gene-Eden-VIR/Novirin versus Acyclovir, Valacyclovir, and Famciclovir. Drug Des. Devel Ther..

[93] Polansky H., Itzkovitz E., Javaherian A. (2016). Clinical Study of Gene-Eden-VIR/Novirin in Genital Herpes: Suppressive Treatment Safely Decreases the Duration of Outbreaks in Both Severe and Mild Cases. Clin. Transl. Med..

